# Pharmacological preconditioning with adenosine A_1_ receptor agonist induces immunosuppression and improves graft survival in novel allogeneic transplantation models

**DOI:** 10.1038/s41598-020-60224-x

**Published:** 2020-03-11

**Authors:** Oshri Naamani, Reut Riff, Cidio Chaimovitz, Julia Mazar, Amos Douvdevani

**Affiliations:** 1 Department of Clinical Biochemistry and Pharmacology, Faculty of Health Sciences, Ben-Gurion University of the Negev and Soroka University Medical Center, Beer-Sheva, Israel; 2 Department of Science, Hemdat Hadarom, College of Education, Netivot, Israel; 3 Department of Nephrology, Faculty of Health Sciences, Ben-Gurion University of the Negev and Soroka University Medical Center, Beer-Sheva, Israel; 4 Laboratory of Hematology, Faculty of Health Sciences, Ben-Gurion University of the Negev and Soroka University Medical Center, Beer-Sheva, Israel

**Keywords:** Allotransplantation, Experimental models of disease

## Abstract

Adenosine is widely known as a potent modulator of innate and acquired immunity. It is released during transplants, and acts on four subtype receptors. In previous studies, we demonstrated that pharmacological preconditioning (PPC), pre-administration of the selective A_1_ receptor (A_1_R) agonist led to A_1_R desensitization, is followed by upregulation of the adenosine A_2A_ receptor. This immunosuppressive effect resulted in lymphopenia, and it reduced T-cell reactivity. The aim of the current study was to challenge the immunosuppressive effects of A_1_R-PPC in models of allogeneic grafts. PPC mice were treated by intraperitoneal injection using specific adenosine A_1_R agonist 24 h and 12 h before starting any procedure. We challenged our method in novel allogeneic muscle and skin grafts models. Mice and grafts were assessed by complete blood counts, MLR from PPC splenocytes, and pathological evaluation. We found a significant reduction in WBC and lymphocyte counts in PPC-treated mice. Two-way MLR with splenocytes from PPC grafted mice showed decreased proliferation and anergy. Histology of PPC allogeneic grafts revealed profoundly less infiltration and even less muscle necrosis compared to vehicle treated allografts. Similar results observed in PPC skin transplantation. To conclude, PPC moderated graft rejection in separate allogeneic challenges, and reduced lymphocytes infiltration and ischemic damage.

## Introduction

Adenosine is widely known as a potent modulator of innate and acquired immunity^1^. Prior data from our group and others shows that ischemia, cell death, and inflammation are associated with adenosine elevation^2–4^. In these pathological conditions, adenosine can either be released from cells or derived from the extracellular enzymatic degradation of ATP by CD39 and CD73^5^. The latter is mainly produced by regulatory T cells (Tregs) where inflammation and immune reactions occur. There are four known types of adenosine G-protein-coupled receptors (GPCR): A_1_ and A_3_ are G_i_ coupled receptors which inhibits adenylyl cyclase activity, leading to a decline in cAMP levels. Conversely, the G_s_ A_2A_ and A_2B_ receptors, which upon activation stimulate adenylyl cyclase, raising cAMP levels^6^.

Adenosine receptors are abundantly expressed on immune and other cells and their signaling reflects the dominant receptor^7^. While A_1_R has the highest affinity for adenosine, A_2A_R was found to be the predominant receptor subtype in immune cells^8^. The seminal study of Ohta and Sitkovsky suggest that A_2A_R, by a negative feedback mechanism, plays a critical role in restriction of inflammation^9^. Since A_1_R – a Gi-coupled receptor and A_2A_R – a Gs-coupled receptor have opposite effects on adenylyl cyclase, the net immunosuppressive activity of A_2A_R is affected by A_1_R signaling. For example, in a mixed lymphocyte reaction (MLR), specific A_1_R agonist activation reverses the A_2A_R agonist inhibitory effect in terms of lymphocyte proliferation and cytokine secretions^10^. In contrast, we have shown that early desensitization of A_1_R can alter the balance towards an immunosuppressive A_2A_R environment^11,12^. In previous studies, we characterized the immunosuppressive effects of A_1_R desensitization by pharmacological preconditioning (PPC)^11^. We found that 24 h pre-activation of the A_1_R by a selective adenosine A_1_R agonist, 2-Chloro-n(6)-cyclopentyladenosine (CCPA), led to downregulation of A_1_R and upregulated A_2A_R, mitigated the inflammatory response against invading bacteria, decreasing the number of blood lymphocytes and their reactivity to mitogen and MLR^13,14^.

In a recent work, we showed that A_1_R elimination by genetic manipulation or by desensitization with PPC, is associated with cAMP elevation and lymphopenia^14^. T-cell dysfunction and lymphocyte apoptosis are known to be linked with cAMP elevation^15–17^. Moreover, Tregs induce suppression in effector T cells either by direct transfer of cAMP via gap junctions^18^, or by PGE_2_^19^ and adenosine secretion^5^.

Organ transplantation is the general treatment for end failure of heart, kidneys, lungs and other essential organs. Suppressing the patient’s natural defense mechanism from rejection of the graft by immunosuppressive drugs is paramount in this procedure. A_2A_R activation was shown to improve transplantation outcome. For example; Lappas *et al*. showed that, during early reperfusion in a lung transplantation model, treatment with an A_2A_R agonist reduced lung inflammation and preserved the pulmonary function^20^. Similarly, Sevigny *et al*. demonstrated enhanced skin allograft transplant survival by activating A_2A_R with specific agonists^21^. We hypothesize that pre- and post-operative modulation of the immune system with adenosine A_1_ receptor agonist, will upregulate the immunosuppressive A_2A_R and improve outcome in transplant recipients.

The aim of the current study was to challenge the immunosuppressive effects of A_1_R-PPC in models of allogeneic grafts.

## Results

### A_1_R receptor reduction affects blood cell lineage

We previously established that adenosine activation downregulates lymphocyte activation both *in vitro* in MLR assays and *in vivo* in sepsis models. We have showed that this downregulation is affiliated to A_1_R pre-activation and its desensitization.

In this study, our aim was to challenge the immunosuppressive effects of A_1_R-PPC in models of allogeneic grafts.

**Figure 1:**
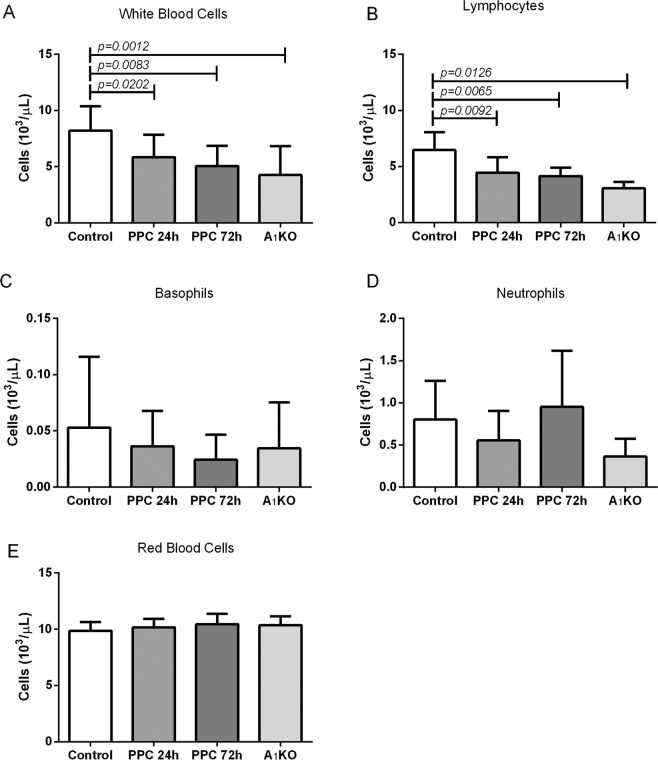
The effect of A_1_ receptor reduction on blood cell lineage. Blood from untreated or 24 h/72 h post-PPC mice or A_1_R-KO mice was collected and analyzed for (**A**) WBC (**B**) Lymphocyte (**C**) Basophils (**D**) Neutrophil and **(E**) Red blood cells counts by an automated differential blood count device (ADIVA 2120). (n = 8–16) Values are mean ± SE.

We conducted blood counts for Vehicle-treated, PPC and A_1_R-KO mice. The latter is used to illustrate the total absence of A_1_R. As shown in Fig. 1, we found a significant reduction in WBC (Fig. 1A) and lymphocyte (Fig. 1B) counts in both PPC-treated and A_1_R-KO mice. The lymphocyte cells were the main cell population affected, with only 3.05 ± 0.4 lymphocytes in A_1_R-KO mice (*p* = 0.012) compared to 6.46 ± 1.52 cells × 10^3^/μl in WT mice – less than 50%. 24 h PPC and 72 h PPC was 4.45 ± 1.29 cells x10^3^/μl (*p* = 0.0202) and 4.14 ± 0.69 cells x10^3^/μl (*p* = 0.083), respectively. Another decrease was also found in basophils and neutrophils at 24 h (Fig. 1C,D respectively). In addition, there were no changes in hematocrit, hemoglobin, mean corpuscular volume (MCV), and mean corpuscular hemoglobin (MCH) at any time course (data not shown).

### A_1_R PPC upregulates A_2A_R

**Figure 2:**
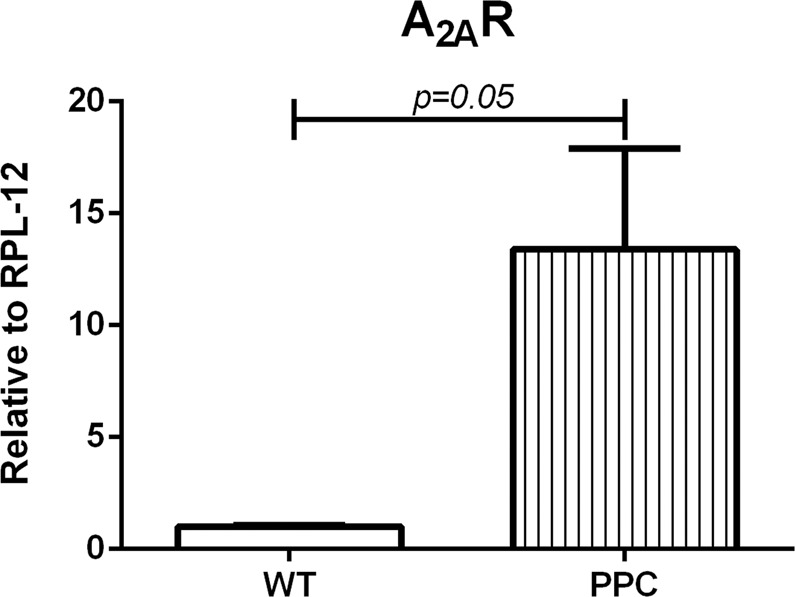
A_1_ receptor reduction affects A_2A_R presence. Mice were administered i.p. with the A_1_R agonist (CCPA, 0.1 mg/kg) or with vehicle. 24 hours later spleen were harvested and cells were incubated in 37 °C for 1 hour. Then adherent cells were scraped and analyzed for A_2A_R mRNA levels. (n = 3).

We determined mRNA levels in the A_2A_R of mice following PPC with the A_1_R agonist (CCPA, 0.1 mg/kg) and found elevation of A_2A_R mRNA levels compared to that of untreated mice (Fig. 2).

### Desensitization of A_1_R restrains leukocyte infiltration and muscle decay *in vivo*

Our previous *in-vitro* results showed that leukocytes from preconditioned mice reduced both proliferation and reactivity.

**Figure 3:**
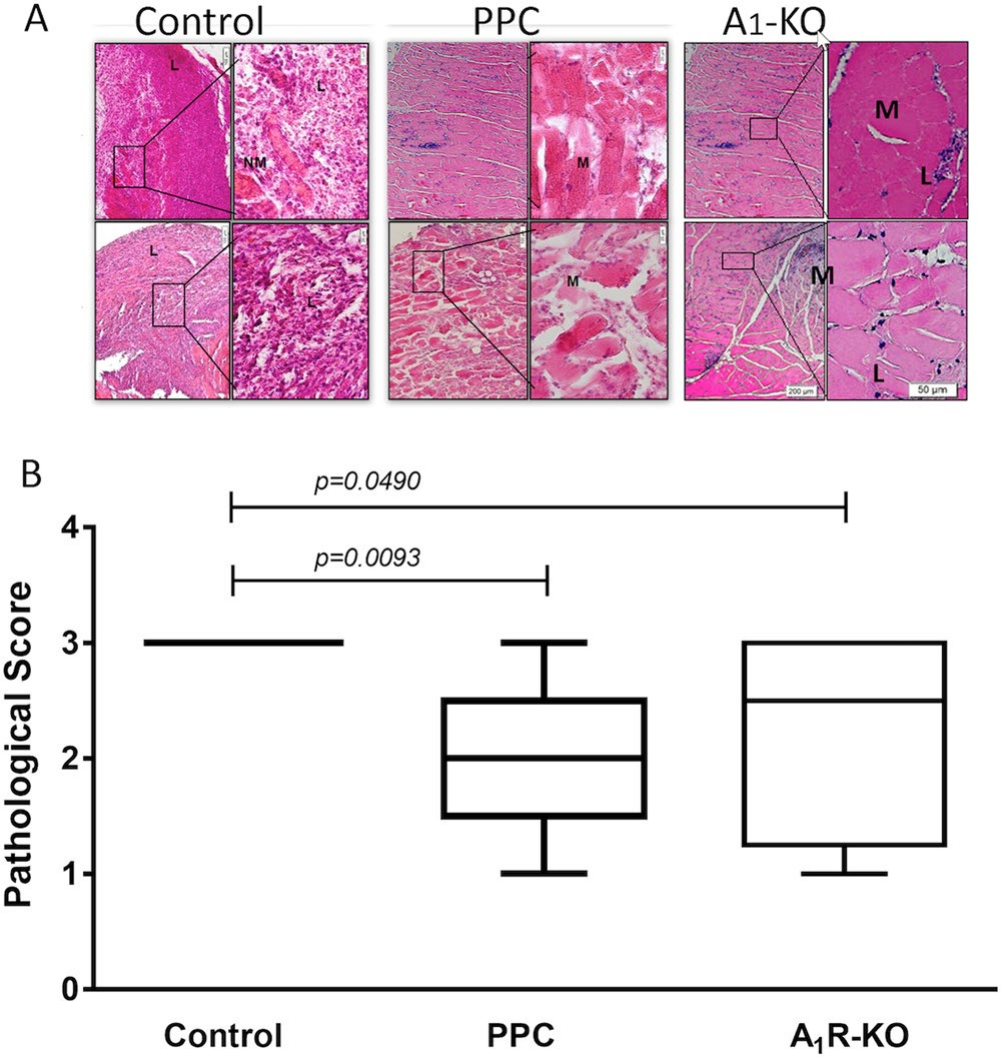
Lack of A_1_ receptor is associated with reduced leukocyte infiltration and necrotic muscle (**A**) and reduced graft decay (**B**) from allogeneic challenged mice on day 10. (**A)** The Pectoralis major muscle, was excised from Balb/c donor mice and grafted in an artificial pocket in the nape of C57 (WT or A_1_R-KO) mice. Recipient mice were PPC with vehicle or with CCPA (0.1 mg/Kg), 24 hours before instillation. The implants were removed on day 10 for further analysis. Grafts were stained with hematoxylin and eosin (H&E) and analyzed for necrosis and leukocyte infiltration. Representative histology images are shown. Scale bar lengths are 200 μm and 50 μm. Arrow indicates nucleus; NM – Necrotic muscle; M – Live muscle; L – lymphocytes. (**B**) Grafts were analyzed and graded for cellular rejection. Data is shown for different recipient mice receiving muscle grafts: Allogeneic n = 9, PPC allogeneic n = 9, A_1_R-KO n = 5.

To evaluate both the leukocytes immunological and ischemic effect *in vivo*, we created an innovative, simple allogeneic graft model. We grafted the *Pectoralis Major* muscle from Balb/c donor mice in an artificial pocket in nape of control C57BL/6 mice. This procedure allows us to evaluate both the infiltrate of leukocytes in the graft and the duration of graft necrosis. The implants were removed on day 10 for further analysis, such as for histological evaluation and scoring. Grafts were graded blindly by a pathologist in an adapted score of ISHLT^22^. Syngeneic muscle grafts shown a transient mild inflammation in the course of 21 days (data not shown). Whether, allogeneic Vehicle-treated muscle histology revealed massive infiltration of medium-to-large atypical leukocytes with both round and irregular nuclei infiltrating T cells, accompanied by infiltrating eosinophils, plasma cells, and neutrophils (Fig. 3A). We also observed blood vessel injury (vasculitis). In addition, there was wide-scale damage to the muscle tissue that matched the state of acute allograft rejection, scored by ISHLT (Fig. 3B). In contrast, PPC muscle histology exposed profoundly less infiltration and less muscle necrosis compared to allografts vehicle-treated mice. Muscles from A_1_R-KO mice, used as a positive control to the A_1_R desensitization by PPC, were also showed moderate signs of inflammation and rejection. The blind grade score confirmed our observation, showing significant differences in favor of PPC and A_1_R-KO muscles (*p* = 0.0093) and (*p* = 0.0490), respectively.

### A_1_R desensitization attenuates MLR proliferation of mice challenged with allogeneic grafts

**Figure 4:**
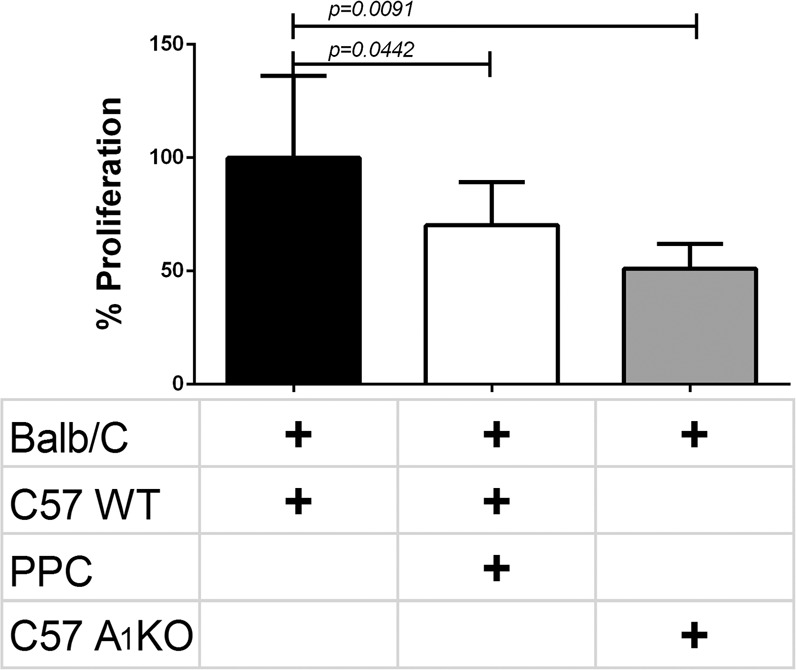
Lack of A_1_ receptor reduced proliferation of splenocyte from allogeneic challenged mice on day 10. The Pectoralis major muscle, was excised from Balb/c donor mice and grafted in an artificial pocket in the nape of C57 (WT or A_1_R-KO) mice. Recipient mice were PPC with vehicle or with CCPA (0.1 mg/Kg), 24 hours before instillation. The implants were removed on day 10 for further analysis. Splenocytes from C57 (2 × 10^5^ cells, responder) mice that underwent allogeneic challenge with Pectoralis Major muscle from Balb/c mice, were stimulated with irradiated Balb/c splenocytes (2 × 10^5^ cells stimulators),10 days post operation for 72 hrs. Treatment included: Control (vehicle treated, n = 9), PPC n = 9, A_1_R-KO n = 5. Values are mean ± SE.

To associate our findings to the above reduction of circulating lymphocytes and alloreactivity, we removed the spleens from the grafted mice. We cultured C57BL/6 responder’s splenocytes from the three groups: Vehicle-treated allogeneic, PPC-treated allogeneic and A_1_R-KO allogenic with stimulators Balb/c splenocytes, in a Two-way MLR. We found that PPC splenocytes that re-encountered allogeneic splenocytes were significantly (*p* = 0.0442) depressed and showed decreased proliferation. A_1_R-KO showed an even greater decrease in proliferation, approximately 50% compared to proliferation in Vehicle-treated allogeneic mice group (Fig. 4).

### PPC attenuates skin allograft rejection

**Figure 5:**
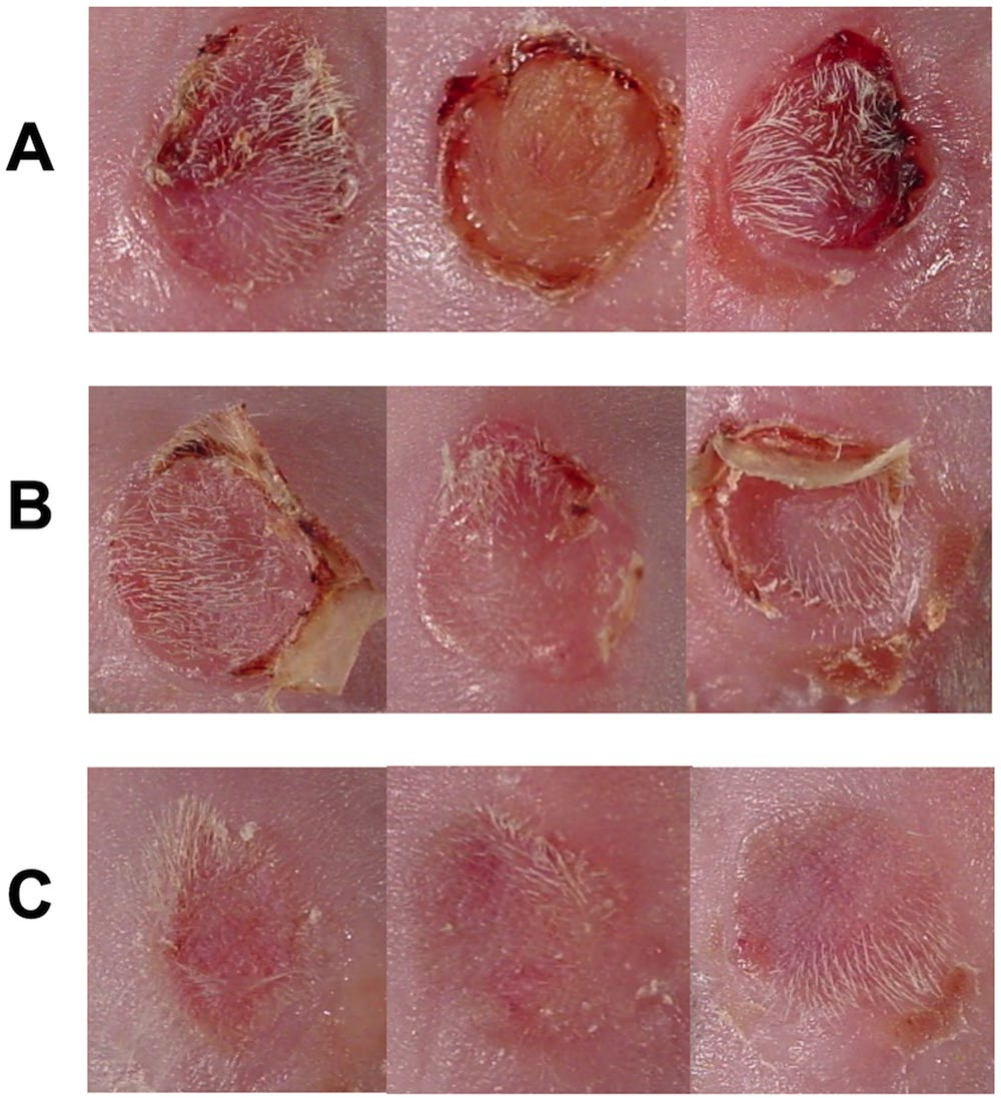
PPC with A_1_R agonist attenuates skin allograft rejection and inflammation early days following transplantation. Representative photographs are shown for recipient mice receiving skin allografts on day 6 (**A**) Vehicle (upper row) and (**B**) PPC with A_1_R agonist CCPA (0.1 mg/kg, middle row). (**C**) Double-side PPC (both the donor and the recipient).

To support our findings on muscle grafts, we also tested the effect of PPC in a skin graft model, in which rejection could be followed continuously without further intervention. In this model, ear skin grafts from Balb/c donor mice were grafted on the dorsal area of vehicle CCPA (0.1 mg/kg)-treated C57BL/6 mice. Graft survival was followed daily by visual inspection and photography from day 5 (removal of bandage) till rejection (loss of all viable skin). Figure 5 shows representative pictures taken at day 6 which illustrate the marked differences between grafts in the vehicle-treated (Fig. 5A) and PPC mice (Fig. 5B). In the vehicle-treated group, we observed clear rejection signs and inflammation such as redness, swelling, and loss of viable skin – effects that were markedly reduced in the pre-conditioned mice. The most significant finding was when PPC was administrated both on the donor and recipient (Fig. 5C).

**Figure 6:**
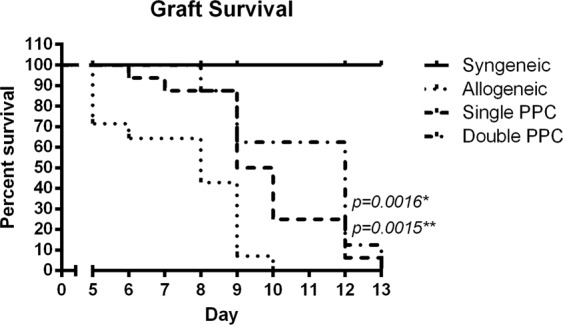
PPC attenuates skin allograft rejection. Ear skin grafts from BALB/c donor mice were grafted on the dorsal area of vehicle-treated syngeneic (N = 5), allogeneic (N = 14), or PPC only to recipient or both donor & recipient (N = 16, N = 8, respectively) C57 mice. Mantel-Cox test for all graft survival. Day 5 indicates removal of bandage. Transplantations were considered as rejected when the graft lost all viable signs. *Between Allogeneic and single PPC ** Between Allogeneic and double PPC.

As shown in Fig. 6, at day five, upon removal of the bandage, significant differences (*p* = 0.0016) were observed between the single PPC group to the vehicle-treated groups. In the vehicle-treated group, only 70% of the allografts remained implanted, while all grafts in the single and double PPC groups were intact. Initial rejection in the PPC group began at day 7 until day 13, when the entire allogeneic population of recipients rejected their grafts with a shift in favor of PPC mice.

## Discussion

Adenosine is a potent modulator of lymphocyte development, proliferation, and activity, and its effect depends both on its bioavailability and on cell surface receptor expression. The role of adenosine signaling in regulating tumor immunity has been widely described for its potential therapeutic role in cancer^23^.

In this study we have shown that modulation of adenosine receptors by A_1_R PPC prolongs allogeneic graft survival, exhibiting lymphocyte blood count reduction and T-cell response moderation.

The PPC protocol was well established in our previous studies^2,13,14^, where we found that stimulation of the pro-inflammatory Gi-coupled receptor A_1_R causes it to become dysfunctional by desensitization, and in parallel subsequentially upregulate the Gs-coupled A_2A_R as the dominant receptor for extracellular adenosine. In a SIRS model, this phenomenon induced leukopenia and reduced T-cell reactivity^14^ and, therefore, led to immunosuppression^2^. These studies led us to test whether PPC could moderate allograft reactions.

It is a common finding that immunosuppressive drugs induce lymphopenia^24–26^. We have shown that PPC significantly reduced lymphocytes in complete blood counts (CBC) in grafted mice. CBC revealed that the white blood cell linage, mainly lymphocyte counts, remained significantly low up to 72 h post-PPC. Lymphocytes are accountable for a majority role in graft rejection elevation, pro-inflammatory cytokines, and stimulation of the immune response^27^. On the other hand, other leukocyte lineages and the red cell linage were not significantly affected by PPC. This specifically induced lymphopenia in mice treated with PPC has important clinical consequences. In addition to reducing T-cytotoxic cells, PPC increased T-regulatory cells and expanded their immunosuppressive effect^13^. For example, Vanasek *et al*. showed that Tregs expanded after lymphopenia and can promote the development of clonal anergy^28,29^.

The elevation of A_2A_R by PPC is critical for the immunosuppressive effect of adenosine. According to Armstrong *et al*., the immunosuppressive response to adenosine is limited by the numbers of A_2A_Rs on T-lymphocytes^30^. Previous studies of immunosuppression in allogeneic transplantation models showed that A_2A_R agonists alone can lead to better acceptance of grafts^21,31,32^. For example, in a murine model of GVHD, Han *et al*. showed that A_2A_R agonists reduced both mortality and the secretion of pro-inflammatory cytokines^33^. However, we believe that dysfunction of A_1_R is, by itself, immunosuppressive, as indicated by our observation that A_1_R-KO mice had a reduced MLR and a moderate reaction towards allogeneic challenge^13^.

For that reason, we established a unique and novel model that allows us to evaluate the immunological and ischemic reaction towards allogeneic grafts. We grafted a thin flap muscle in the nape of allogenic matched strain, in a way that we can excised the flap and examined its viability and alloreactivity. This model can be easily applied to various pharmacological or anti-inflammatory treatments.

We have blindly and significantly showed that the treatment groups were either PPC 24 h before instillation or were A_1_R-KO mice were better preserved and exhibited fewer infiltrating cells compared to vehicle-treated mice. Furthermore, splenocytes from the grafted mice that were exposed for 10 days to allogeneic implants, were challenged for MLR. It is known that low MLR response is predictive to successful transplantation^34–36^. We have shown that a single PPC treatment effectively and significantly reduced lymphocyte response. This anergic effect in accordance with our previous findings indicating that the suppression in MLR is associated with an elevation of A_2A_R following PPC. Similar results are also shown when A_2A_R agonist decreased proliferation in allogeneic MLR assays^10,21^.

This PPC imprinting process, by which a brief stimulation period of the lymphocytes *in vivo* establishes a long-lasting depressed response is a new strategy and can be easily applied. This phenomenon of a continued response was previously shown by Koshiba *et al*. and named the “memory” of T cells, which suggests that brief exposure of T cells to adenosine *in vitro* is sufficient to observe the inhibition of TCR-triggered effector functions^37^.

Finally, we challenged the PPC method in the skin allograft model. For this evaluation, we managed to establish a skin grafting model that had the advantages of being relatively easy to reproduce and not requiring sutures. When we conducted syngeneic transplantation, all five mice remained alive with the graft up to 8 months post transplantation. We showed that PPC treatment significantly attenuated skin graft rejection compared to vehicle-treated grafts. In addition we noticed that when PPC was administered to both the donor and recipient, we were able to propone the course of rejection in the early days after bandage removal.

The first hours post-graft implantation are critical for survival. The stressed ischemic tissue induces the release of ROS, cytokines, chemokines, and adenosine^38,39^. Activation of adenosine A_2A_R has already been shown to have a protective effect during liver transplantation^40^. Therefore, it is possible that this effect could be intensified with PPC, allowing endogenous extracellular adenosine to act mainly on A_2A_R, without the contradictory effects of A_1_R.

In conclusion, we have demonstrated that PPC moderates graft rejection. We believe that A_1_R activation, followed by its desensitization and induction of A_2A_R, shifts the pro-inflammatory danger signal of extracellular adenosine in the graft milieu to an anti-inflammatory response.

Considering the minimal side effects of this treatment, this approach is relevant to the recipient, as well as to healthy or brain-dead live donors. We believe that PPC can be integrated as a pre-transplantation preparation in the future, along with the concept of treating both the donor and the recipient, thereby improving the treatment.

## Materials and Methods

### Mice

All the experimental protocols including operations and postoperative procedures were conducted after obtaining permission from the Israel Committee for Animal Experiments (IL-01-01-2009, IL-24-04-12). All experiments were approved and performed in accordance with relevant guidelines and regulations by the Ben-Gurion University Committee for Ethical Care and Use of Animals in Experiments.

BALB/c and C57BL/6 mice were purchased from Harlan (Jerusalem, Israel), and A_1_R-Knockout mice (A_1_R-KO on C57BL/6 background) were purchased from the Jackson Laboratory (Bar Harbor, ME, USA). Mice were housed and maintained under specific conditions in the vivarium of Ben-Gurion University.

### Pharmacological preconditioning (PPC)

We treated mice as previously described^11,13^ In brief, for PPC, mice were treated by intraperitoneal injection (i.p.) using 2-chloro-N6-cyclopentyladenosine (CCPA 0.1 mg/kg), a specific adenosine A_1_R agonist, 24 h and 12 h before conducting any of the listed below procedures.

### Differential blood cell counts

Blood samples were counted with an ADIVA 2120 blood count device (Siemens; Munich, Germany).

### mRNA analysis by quantitative PCR

24 h after PPC spleens were removed and cells were isolated and treated with a red blood cell (RBC) lysis solution (5 Prime Inc.; Gaithersburg, MD, USA). Cells were incubated for 1 h, and adhesion cells were collected. PerfectPure RNA Tissue Kit (5 Prime Inc.) was used to extracted the RNA. High capacity cDNA reverse transcription kit (Applied Biosystems; Foster City, CA, USA) was used to prepare cDNA. Quantitative real-time polymerase chain reaction (qPCR) assays were performed with a Fast SYBR Green Master Mix (Applied Biosystems) on a StepOne Plus real-time PCR machine (Applied Biosystems).cDNA specific primers were used for A_2A_R quantity: sense 5′-CGC AGG TCT TTG TGG AGT TC-3′, anti-sense 5′-TGG CTT GGT GAC GGG TATG-3′. For reference gene we used RPL-12: sense 5′-ATG ACA TTG CCA AGG CTA CC-3′, anti-sense 5′-CAA GAC CGG TGT CTC ATC TGC -3′.

### Transplantation models

Transplantations were always conducted between C57BL/6 (recipient) WT or A_1_R-KO and Balb/c female (donor) mice (7 wks of age, Harlan; Jerusalem, Israel). Mice were allowed free access to food and water. Experiments were performed on 7- to 11-week-old mice.

### Muscle allogeneic challenge model

In order to evaluate the immunological and ischemic reaction of PPC in allografts we established a simple, easy to reproduce, novel model of allogeneic graft. We performed a small, sterile incision in the nape of recipient mice. To this artificial pocked we grafted the *Pectoralis Major* muscle from donor mice. Due to his thin structure the diffusion of nutrients and oxygen is effective and the ischemic stress is minimal. This procedure allows us to remove the graft in any time point, easily without damage to the recipient mice. The grafts were then sent for histological and immunological evaluation as described below.

#### Recovery of organs

Grafts were removed, and biopsies of the parietal muscle were fixed and stained with hematoxylin and eosin (H&E). Grafts were graded blindly by our pathologist in an adapted ISHLT score^22^. In brief, the revised categories of cellular rejection were as follows: Grade 0 – no rejection, Grade 1 – mild rejection, Grade 2 – moderate rejection, and Grade 3 – severe rejection.

#### Isolation of mononuclear cells from spleens

As we described earlier^14^, spleens were removed from mice and disrupted under sterile conditions in phosphate buffer saline (PBS) through 40-μm BD Falcon cell strainers (Fisher Scientific; Pittsburgh, PA, USA). Mononuclear cells were then isolated via density gradient centrifugation using Histopaque 1083 (Sigma-Aldrich). Cells were washed twice, and total leukocytes were counted after trypan blue staining using an improved Neubaur hemocytometer. Cells were grown in RPMI 1640 medium and supplemented with 10% heat-inactivated fetal calf serum (FCS), 2 mmol/l L-glutamine, 100 U/ml penicillin, and 100 μg/ml streptomycin (Biological Industries; Bet Haemek, Israel).

#### Activation of leukocytes

Leukocyte activation was performed using 96-well flat-bottom culture plates (Greiner Bio-One; Germany) for 72 h at 37 °C in the presence of 5% CO2. For standard two-way MLR assays, responder cells (total splenocytes, 2 × 10^5^) were co-cultured with an equal number of stimulator splenocytes in 200 μl medium^14,16^. Thymidine (1 μ Ci/well; PerkinElmer Life and Analytical Sciences) was added 18–24 h before recovering (Inotech Biosystems International Inc.) using Type A filter mats (PerkinElmer Life and Analytical Sciences) and a beta-plate scintillation mixture (PerkinElmer). CPM were determined using a liquid scintillation analyzer (Packard 1900CA, Packard Instrument Co.). Data were expressed as the mean CPM of triplicate determination, and converted into proliferation percentages. 100% proliferation refers to vehicle treated allogeneic group. Splenocytes background readout values (medium alone) were deducted from the results.

### Skin transplant procedure

For skin grafts we used half-thickness ear skin (~0.7 cm^2^) that were from donor mice, and were grafted on the dorsal area of the recipient mice. After the procedure, the grafts were wrapped in a sterile bandage (with the non-adhesive gauze segment placed over the skin graft), and tied loosely enough to allow for breathing and free arm mobility. Recipient mice were monitored daily for any signs of distress, and an analgesic was administered if needed for pain relief. Mice were anesthetized using the above procedure, and the bandages were cut and removed using blunt-end scissors. Grafts were checked in the first hours for signs of scabbing or contraction. If present, then grafts did not vascularize and were considered to be failures. Grafts were monitored daily for signs of rejection (usually defined as ~80% necrosis of the donor tissue).

To asses graft survival we performed daily recordings from day 6 to day 9. Later on we analyzed it by visual inspection in a masked fashion.

### Statistical analysis

The comparisons was carried out using one of the following: a Mann–Whitney nonparametric t-test or by a one-way ANOVA followed by a Tukey post-test. All comparison were preform using Graphpad Prism 5 software (GraphPad; San Diego, CA). Survival grafts were analyzed by Mantel-Cox test. *P* values below 0.05 were considered significant. Values are presented as mean ± SEM.

## Data Availability

The datasets generated during and/or analyzed during the current study are available from the corresponding author on reasonable request.

## References

[1] Cekic C, Linden J (2016). Purinergic regulation of the immune system. Nat Rev Immunol.

[2] Nakav S (2010). Regulation of adenosine system at the onset of peritonitis. Nephrol Dial Transplant.

[3] Martin C, Leone M, Viviand X, Ayem ML, Guieu R (2000). High adenosine plasma concentration as a prognostic index for outcome in patients with septic shock. Crit Care Med.

[4] Gruber HE (1989). Increased adenosine concentration in blood from ischemic myocardium by AICA riboside. Effects on flow, granulocytes, and injury. Circulation.

[5] Deaglio S (2007). Adenosine generation catalyzed by CD39 and CD73 expressed on regulatory T cells mediates immune suppression. J Exp Med.

[6] Hasko G, Cronstein B (2013). Regulation of inflammation by adenosine. Front Immunol.

[7] Hasko G, Linden J, Cronstein B, Pacher P (2008). Adenosine receptors: therapeutic aspects for inflammatory and immune diseases. Nat Rev Drug Discov.

[8] Fredholm BB (2007). Adenosine, an endogenous distress signal, modulates tissue damage and repair. Cell Death Differ.

[9] Ohta A, Sitkovsky M (2001). Role of G-protein-coupled adenosine receptors in downregulation of inflammation and protection from tissue damage. Nature.

[10] Takahashi Hideo Kohka, Iwagaki Hiromi, Hamano Ryosuke, Kanke Toru, Liu Keyue, Sadamori Hiroshi, Yagi Takahito, Yoshino Tadashi, Sendo Toshiaki, Tanaka Noriaki, Nishibori Masahiro (2007). Effect of adenosine receptor subtypes stimulation on mixed lymphocyte reaction. European Journal of Pharmacology.

[11] Nakav S (2008). Anti-inflammatory preconditioning by agonists of adenosine A1 receptor. PLoS One.

[12] Nakav S (2009). Blocking adenosine A2A receptor reduces peritoneal fibrosis in two independent experimental models. Nephrol Dial Transplant.

[13] Naamani O, Chaimovitz C, Douvdevani A (2014). Pharmacological preconditioning with adenosine A(1) receptor agonist suppresses cellular immune response by an A(2A) receptor dependent mechanism. Int Immunopharmacol.

[14] Riff R (2017). Systemic inflammatory response syndrome-related lymphopenia is associated with adenosine A1 receptor dysfunction. J Leukoc Biol.

[15] Koshiba M, Rosin DL, Hayashi N, Linden J, Sitkovsky MV (1999). Patterns of A2A extracellular adenosine receptor expression in different functional subsets of human peripheral T cells. Flow cytometry studies with anti-A2A receptor monoclonal antibodies. Mol Pharmacol.

[16] Zarek PE (2008). A2A receptor signaling promotes peripheral tolerance by inducing T-cell anergy and the generation of adaptive regulatory T cells. Blood.

[17] Wilson JM (2011). The A2B adenosine receptor promotes Th17 differentiation via stimulation of dendritic cell IL-6. J Immunol.

[18] Bopp T (2007). Cyclic adenosine monophosphate is a key component of regulatory T cell-mediated suppression. J Exp Med.

[19] Mahic M, Yaqub S, Johansson CC, Tasken K, Aandahl EM (2006). FOXP3+CD4+CD25+ adaptive regulatory T cells express cyclooxygenase-2 and suppress effector T cells by a prostaglandin E2-dependent mechanism. J Immunol.

[20] Lappas CM, Day YJ, Marshall MA, Engelhard VH, Linden J (2006). Adenosine A2A receptor activation reduces hepatic ischemia reperfusion injury by inhibiting CD1d-dependent NKT cell activation. J Exp Med.

[21] Sevigny CP (2007). Activation of adenosine 2A receptors attenuates allograft rejection and alloantigen recognition. J Immunol.

[22] Lappas CM, Rieger JM, Linden J (2005). A2A adenosine receptor induction inhibits IFN-gamma production in murine CD4+ T cells. J Immunol.

[23] Leone RD, Emens LA (2018). Targeting adenosine for cancer immunotherapy. J Immunother Cancer.

[24] Gergely P (1999). Drug-induced lymphopenia: focus on CD4+ and CD8+ cells. Drug Saf.

[25] Noris Marina, Casiraghi Federica, Todeschini Marta, Cravedi Paolo, Cugini Daniela, Monteferrante Giuseppe, Aiello Sistiana, Cassis Linda, Gotti Eliana, Gaspari Flavio, Cattaneo Dario, Perico Norberto, Remuzzi Giuseppe (2007). Regulatory T Cells and T Cell Depletion: Role of Immunosuppressive Drugs. Journal of the American Society of Nephrology.

[26] Pepper, A. N., Talreja, N., Cowan, G. M., Glaum, M. C. & Lockey, R. F. Lymphopenia induced by etanercept. *Ann Allergy Asthma Immunol* **112**, 262–263, S1081-1206(13)00949-6 (2014). 10.1016/j.anai.2013.12.019 24565596

[27] Krensky AM, Weiss A, Crabtree G, Davis MM, Parham P (1990). T-lymphocyte-antigen interactions in transplant rejection. N Engl J Med.

[28] Minamimura, K., Gao, W. & Maki, T. CD4+ regulatory T cells are spared from deletion by antilymphocyte serum, a polyclonal anti-T cell antibody. *J Immunol* **176**, 4125-4132, 176/7/4125 (2006). 10.4049/jimmunol.176.7.4125 16547248

[29] Vanasek, T. L., Nandiwada, S. L., Jenkins, M. K. & Mueller, D. L. CD25+Foxp3+ regulatory T cells facilitate CD4+ T cell clonal anergy induction during the recovery from lymphopenia. *J Immunol* **176**, 5880-5889, 176/10/5880 (2006). 10.4049/jimmunol.176.10.5880 16670295

[30] Armstrong JM (2001). Gene dose effect reveals no Gs-coupled A2A adenosine receptor reserve in murine T-lymphocytes: studies of cells from A2A-receptor-gene-deficient mice. The Biochemical journal.

[31] Sahara H (2018). Induction of Tolerance to Fully Allogeneic Pulmonary Allograft by Adenosine A2A Receptor Agonist in MHC-defined CLAWN Miniature Swine. Transplantation.

[32] Lappas CM, Liu PC, Linden J, Kang EM, Malech HL (2010). Adenosine A2A receptor activation limits graft-versus-host disease after allogenic hematopoietic stem cell transplantation. J Leukoc Biol.

[33] Han KL (2013). Adenosine A(2)A receptor agonist-mediated increase in donor-derived regulatory T cells suppresses development of graft-versus-host disease. J Immunol.

[34] Kerman R.H., Katz S.M., Schoenberg L., Baraket O., Van Buren C.T., Kahan B.D. (1997). Ten-year follow-up of mixed lymphocyte reaction-hyporesponsive living related cyclosporine monotherapy-treated renal allograft recipients. Transplantation Proceedings.

[35] Le Moine A, Goldman M, Abramowicz D (2002). Multiple pathways to allograft rejection. Transplantation.

[36] Cosmi L (2004). Th2 cells are less susceptible than Th1 cells to the suppressive activity of CD25+ regulatory thymocytes because of their responsiveness to different cytokines. Blood.

[37] Koshiba M, Kojima H, Huang S, Apasov S, Sitkovsky MV (1997). Memory of extracellular adenosine A2A purinergic receptor-mediated signaling in murine T cells. J Biol Chem.

[38] Dale N, Frenguelli BG (2009). Release of adenosine and ATP during ischemia and epilepsy. Curr Neuropharmacol.

[39] Kalogeris T, Baines CP, Krenz M, Korthuis RJ (2012). Cell biology of ischemia/reperfusion injury. Int Rev Cell Mol Biol.

[40] Tang LM (2007). Protective effect of adenosine A2A receptor activation in small-for-size liver transplantation. Transpl Int.

